# The combined importance of finite dimensions, anisotropy, and pre-stress in acoustoelastography

**DOI:** 10.1121/10.0010110

**Published:** 2022-04-07

**Authors:** Joseph Crutison, Michael Sun, Thomas J. Royston

**Affiliations:** 1 Richard and Loan Hill Department of Biomedical Engineering, University of Illinois Chicago, 851 South Morgan Street, MC 063, Chicago, Illinois 60607, USA; 2 Department of Ophthalmology and Visual Sciences, University of Illinois Chicago, Chicago, Illinois 60612, USA

## Abstract

Dynamic elastography, whether based on magnetic resonance, ultrasound, or optical modalities, attempts to reconstruct quantitative maps of the viscoelastic properties of biological tissue, properties that are altered by disease and injury, by noninvasively measuring mechanical wave motion in the tissue. Most reconstruction strategies that have been developed neglect boundary conditions, including quasistatic tensile or compressive loading resulting in a nonzero prestress. Significant prestress is inherent to the functional role of some biological tissues currently being studied using elastography, such as skeletal and cardiac muscle, arterial walls, and the cornea. In the present article, we review how prestress alters both bulk mechanical wave motion and wave motion in one- and two-dimensional waveguides. Key findings are linked to studies on skeletal muscle and the human cornea, as one- and two-dimensional waveguide examples. This study highlights the underappreciated combined acoustoelastic and waveguide challenge to elastography. Can elastography truly determine viscoelastic properties of a material when what it is measuring is affected by both these material properties and unknown prestress and other boundary conditions?

## I. INTRODUCTION

### A. Background and motivation

Dynamic elastography methods–based on optical, ultrasonic, and magnetic resonance imaging modalities–aim to quantitatively map the shear viscoelastic properties of biological tissue, which are often altered by disease and injury. Optical methods, including early work using stroboscopy^1^ to more recent, higher resolution methods using optical coherence tomography^2,3^ or laser Doppler vibrometry,^4^ have the advantage of the highest resolution of the three modalities but are constrained to the penetration depth of light, limiting their clinical use to measurements at or near the surface, such as the skin or cornea. Ultrasound (US)-based elastography using continuous or transient excitation has greater penetration depth and maintains high resolution near the surface (though not as high as optical methods), while also being readily available, contributing to its wide use in research since the late 1980s.^5–9^ Magnetic resonance elastography (MRE), introduced in 1995,^10^ has the highest depth of penetration, even behind hard tissue obstacles, such as the skull, and the ability to encode oscillatory motion in all three dimensions simultaneously;^11^ but it is limited in resolution as compared to optical methods and near-surface US methods and is the most expensive, as compared to all other modalities. Regardless of the imaging modality, dynamic elastography methods share common traits. They typically involve mechanical stimulation followed by measurement and analysis of resulting transverse wave motion in order to estimate or reconstruct the tissue's shear viscoelastic properties.

Most initial studies focused on larger organs, such as the liver or brain, where boundary effects were assumed negligible. But as elastography expands to other anatomical regions where dimensions in at least one direction are smaller or of comparable length to bulk shear wavelengths–such as in slender skeletal muscles, blood vessels, the heart wall, and the cornea–boundary effects become non-negligible and must be considered. Researchers using optical coherence elastography (OCE) to assess the viscoelastic properties of the cornea have long recognized this, adapting models to include waveguides by treating the cornea as a plate-like structure that is fluid-loaded on one side. Here, transverse wave motion on the cornea is modeled as Rayleigh–Lamb waves.^3^ Blood vessels, as well, have been modeled using cylindrical shell equations considering fluid–structure interaction.^12–16^ Limited studies on cardiac elastography have also acknowledged the frequency-dependent (i.e., wavelength-dependent) waveguide behavior of the heart wall.^17^

Application of dynamic elastography to tissues with aligned fibrous structure resulting in local *transverse isotropic* mechanical properties, such as can be found in striated skeletal and cardiac muscle, as well as brain white matter, may benefit from analysis that takes into consideration anisotropy of the tissue. Recognizing this, some groups have pioneered research in this direction over the past few decades, using ultrasound (US)-based elastography,^9,18–20^ as well as magnetic resonance (MR)-based elastography.^21,22^ Many of these studies have tried to tackle the associated inversion problem. Multiple configurations or a multi-directional shear wave excitation source may be needed in order to generate and measure shear wave motion that will be affected by its displacement polarization direction and propagation direction in an anisotropic material.

In addition to a uniaxially aligned fibrous structure, a layered nonfibrous or fibrous structure, such as in the cornea, can also have a significant effect on shear wave behavior. The cornea can be considered as a transversely isotropic material where the axis of isotropy is perpendicular to the plane of the cornea.^23^ Here, it has been shown that both an in-plane and out-of-plane shear moduli are required to fully describe the elastic properties of corneal tissue. This important observation has provided a possible explanation for the differences in magnitude seen with elastic moduli measured using dynamic elastography techniques compared to traditional uniaxial tensile testing of cornea tissue.

Often, when elastography studies are done under varying quasi-static *pre-stress* conditions, observed changes in mechanical wave behavior are attributed solely to the nonlinear property of the tissue: it has been suggested that its shear and viscous constants are highly dependent on the tensile load and associated deformation. In previous muscle elastography studies, it has been surmised that the shear elastic modulus increased with passive muscle loading^24–30^ or with muscle activation.^22,26,29,31–34^ In other studies, it is simply observed that the shear wave velocity increased with increased passive or active loading of the muscle.^35^ While the latter is an indisputable observation–the velocity increased with increased load–the former (change in moduli value) ascribes the wavelength change solely to a change in tissue material properties, which we propose may or may not be responsible for a fraction of the observed changes in measured transverse wave speed.

Some have recognized the influence of compressive forces on elastography measurements of shear waves on phantoms and organs.^36–38^ An early study^39^ using MR elastography recognized that both tensile load and material elastic moduli affected transverse wave motion in skeletal muscle under tension. This was an *in vivo* study of the tibialis anterior (TA) and lateral gastrocnemius (LG), showing that shear wavelength increased when the muscle was stretched and when the muscle was contracted. In the Discussion section, a linear equation was put forth to account for both tensile and shear modulus effects, as an explanation for the observed changes. A more recent MR elastography study^40^ made a similar observation but replaced the bulk shear wave expression with one based on a Timoshenko beam under tension formulation; thus, also accounting for waveguide effects, and implemented this in an inversion strategy to assess tensile forces on the individual muscles of the forearm.

### B. Objectives

In the present study, we review the theoretical principles of mechanical wave motion in a normally prestressed transversely isotropic material, and then consider two- and one-dimensional “thin” waveguides subjected to tensile loading, relating fundamental observations to applications in cornea and muscle elastography. Some of the presented concepts, without the supporting analyses detailed here, were previously summarized by the last author in an invited abstract and oral presentation at a recent meeting of the Acoustical Society of America.^41^

## II. PRE-STRESS UNDER SMALL DEFORMATION IN A TRANSVERSE ISOTROPIC MATERIAL

Building upon Tweten *et al.*,^42,43^ we start with a *linear elastic nearly incompressible, transversely isotropic (NITI)* material as our model for biological tissue with aligned fibrous structure subjected to deformation that is sufficiently small in amplitude to justify the assumption of linearity. A linear elastic NITI material may be fully described using bulk modulus κ and three additional parameters which can be a combination of two tensile moduli, $E_{⊥}\;\;\mathrm{and}\;\;E_{∥},$ and two shear moduli, $\mu_{⊥}\;\;\mathrm{and}\;\;\mu_{∥}$, where the subscripts denote whether the principle direction is perpendicular or parallel to the fiber direction. In other words, $E_{⊥}\;\;\mathrm{and}\;\;\mu_{⊥}$ are in the direction perpendicular to the fibers (parallel to the plane of isotropy), and $E_{∥}\;\;\mathrm{and}\;\;\mu_{∥}$ are in the direction parallel to the fibers (parallel to the axis of isotropy). We define shear anisotropy $\phi =\mu_{∥}/\mu_{⊥}-1$ and tensile anisotropy $\zeta =E_{∥}/E_{⊥}-1$. Note also that^44^ $E_{∥}=\mu_{⊥}\left(4\zeta +3\right)$; thus, there are only three independent parameters.

The transverse isotropic elasticity matrix K using nomenclature from Guidetti *et al.*^45^ and taking the z-axis at the axis of isotropy is

$$\begin{array}{c} \left[\begin{array}{c} \sigma_{xx}\\ \sigma_{yy}\\ \sigma_{zz}\\ \sigma_{yz}\\ \sigma_{xz}\\ \sigma_{xy} \end{array}\right]=\left[\begin{array}{c} \begin{array}{cccccc} \kappa +\mu_{⊥}\left(\frac{4}{3}+\frac{4}{9}\zeta \right) & \kappa -\mu_{⊥}\left(\frac{2}{3}-\frac{4}{9}\zeta \right) & \kappa -\mu_{⊥}\left(\frac{2}{3}+\frac{8}{9}\zeta \right) & 0 & 0 & 0\\ \kappa -\mu_{⊥}\left(\frac{2}{3}-\frac{4}{9}\zeta \right) & \kappa +\mu_{⊥}\left(\frac{4}{3}+\frac{4}{9}\zeta \right) & \kappa -\mu_{⊥}\left(\frac{2}{3}+\frac{8}{9}\zeta \right) & 0 & \;0 & \;0\\ \kappa -\mu_{⊥}\left(\frac{2}{3}+\frac{8}{9}\zeta \right) & \kappa -\mu_{⊥}\left(\frac{2}{3}+\frac{8}{9}\zeta \right) & \kappa +\mu_{⊥}\left(\frac{4}{3}+\frac{16}{9}\zeta \right) & 0 & \;0 & \;0\\ 0 & \;0 & \;0 & \mu_{⊥}\left(1+\phi \right) & 0 & 0\\ 0 & \;0 & \;0 & 0 & \mu_{⊥}\left(1+\phi \right) & 0\\ 0 & \;0 & \;0 & 0 & 0 & \mu_{⊥} \end{array} \end{array}\right]\left[\begin{array}{c} \varepsilon_{xx}\\ \varepsilon_{yy}\\ \varepsilon_{zz}\\ {2\varepsilon }_{yz}\\ {2\varepsilon }_{xz}\\ {2\varepsilon }_{xy} \end{array}\right]. \end{array}$$ (1)

Adding only normal (no shear) static pre-stresses $\sigma_{x},\;\sigma_{y},\;\mathrm{and}\;\sigma_{z}$ aligned with the x, y, and z directions, respectively, leads to the following governing equations of motion,^46,47^ where *u*, *v*, and *w* refer to the displacement component in the x, y, and z direction, respectively, and subscripted x, y, z, and *t* after a comma refer to partial derivatives with respect to that spatial or time dimension:

$$\begin{array}{c} \rho u_{,tt}=\left(\kappa +\mu_{⊥}\left(\frac{4}{3}+\frac{4}{9}\zeta \right)\right)u_{,xx}+\left(\mu_{⊥}-\frac{\sigma_{x}-\sigma_{y}}{2}\right)u_{,yy}+\left(\mu_{⊥}\left(1+\phi \right)+\frac{\sigma_{z}-\sigma_{x}}{2}\right)u_{,zz}\\ +\left(\kappa +\mu_{⊥}\left(\frac{1}{3}+\frac{4}{9}\zeta \right)+\frac{\sigma_{x}-\sigma_{y}}{2}\right)v_{,xy}+\left(\kappa +\mu_{⊥}\left(\frac{1}{3}+\phi -\frac{8}{9}\zeta \right)+\frac{\sigma_{x}-\sigma_{z}}{2}\right)w_{,xz}, \end{array}$$ (2)

$$\begin{array}{c} \rho v_{,tt}=\left(\kappa +\mu_{⊥}\left(\frac{4}{3}+\frac{4}{9}\zeta \right)\right)v_{,yy}+\left(\mu_{⊥}+\frac{\sigma_{x}-\sigma_{y}}{2}\right)v_{,xx}+\left(\mu_{⊥}\left(1+\phi \right)+\frac{\sigma_{z}-\sigma_{y}}{2}\right)v_{,zz}\\ +\left(\kappa +\mu_{⊥}\left(\frac{1}{3}+\frac{4}{9}\zeta \right)+\frac{\sigma_{y}-\sigma_{x}}{2}\right)u_{,xy}+\left(\kappa +\mu_{⊥}\left(\frac{1}{3}+\phi -\frac{8}{9}\zeta \right)+\frac{\sigma_{y}-\sigma_{z}}{2}\right)w_{,yz}, \end{array}$$ (3)

$$\begin{array}{c} \rho w_{,tt}=\left(\kappa +\mu_{⊥}\left(\frac{4}{3}+\frac{16}{9}\zeta \right)\right)w_{,zz}+\left(\mu_{⊥}\left(1+\phi \right)+\frac{\sigma_{y}-\sigma_{z}}{2}\right)w_{,yy}+\left(\mu_{⊥}\left(1+\phi \right)+\frac{\sigma_{x}-\sigma_{z}}{2}\right)w_{,xx}\\ +\left(\kappa +\mu_{⊥}\left(\frac{1}{3}+\phi -\frac{8}{9}\zeta \right)+\frac{\sigma_{z}-\sigma_{x}}{2}\right)u_{,xz}+\left(\kappa +\mu_{⊥}\left(\frac{1}{3}+\phi -\frac{8}{9}\zeta \right)+\frac{\sigma_{z}-\sigma_{y}}{2}\right)v_{,yz}. \end{array}$$ (4)

Consider plane wave propagation in the x, y, and z directions. Based on Eqs. (2)–(4) phase speeds for propagation in these three directions with two possible polarizations for shear waves, and one polarization for compression waves, is provided in Table I. (Note, this formulation, based inherently on a small strain assumption due to the applied stress and linear elastic theory, can be made to match predictions of phase speed in isotropic^26,48^ and transverse isotropic^49^ acoustoelastic models that allow for larger strain values by appropriate choice of third order Landau coefficients. For the isotropic case, this is achieved by setting the third order coefficient A=−6μ, which is within the range of values reported for agar-gelatin and polyvinyl alcohol soft tissue phantom materials based on ultrasound elastography measurements under compressive stress loading. For the transverse isotropic case, three additional third order Landau coefficients^49^ are set to specific values to match the small strain assumption used here. See the Appendix for additional explanation.)

**TABLE I.** Phase speed squared * density $\left(\rho c^{2}\right)$ of planar waves as function of propagation and polarization directions.

Polarization →	x direction	y direction	z direction
↓ Propagation			
x direction	$\kappa +\mu_{⊥}\left(\frac{4}{3}+\frac{4}{9}\zeta \right)$	$\mu_{⊥}+\frac{\sigma_{x}-\sigma_{y}}{2}$	$\mu_{⊥}\left(1+\phi \right)+\frac{\sigma_{x}-\sigma_{z}}{2}$
y direction	$\mu_{⊥}+\frac{\sigma_{y}-\sigma_{x}}{2}$	$\kappa +\mu_{⊥}\left(\frac{4}{3}+\frac{4}{9}\zeta \right)$	$\mu_{⊥}\left(1+\phi \right)+\frac{\sigma_{y}-\sigma_{z}}{2}$
z direction	$\mu_{⊥}\left(1+\phi \right)+\frac{\sigma_{z}-\sigma_{x}}{2}$	$\mu_{⊥}\left(1+\phi \right)+\frac{\sigma_{z}-\sigma_{y}}{2}$	$\kappa +\mu_{⊥}\left(\frac{4}{3}+\frac{16}{9}\zeta \right)$

## III. ADDING BOUNDARY EFFECTS IN THE 3-DIMENSIONAL PROBLEM

**FIG. 1.**
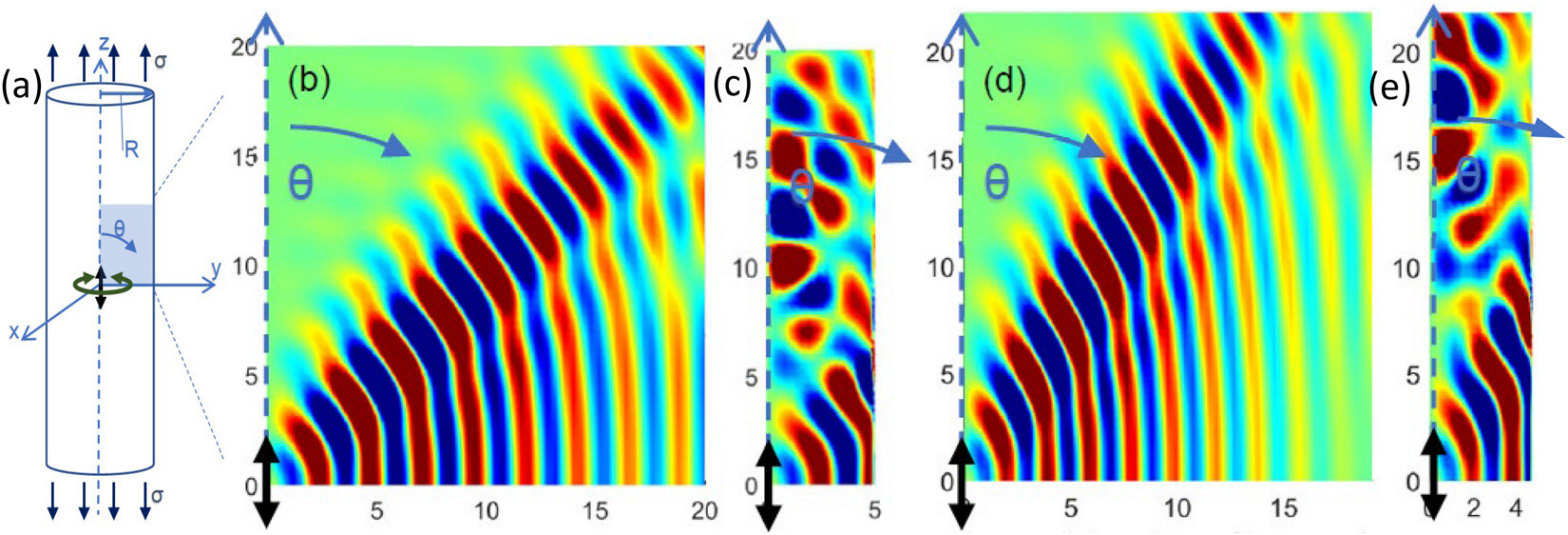
(Color online) Fast shear waves in the y-z plane generated by a harmonic point force at the origin polarized parallel to the axis of isotropy (z-axis) and direction of uniaxial stress σ, using material properties from our previous work (Refs. 45 and 51–53) that correspond approximately to passive muscle with fibers along the z-axis (Table II). Simulations are for no pre-stress (b) and (c) or a uniaxial prestress from elongation along fiber direction that is equivalent to the stress from a 10% MVC contraction (d) and (e). All axes in (b) and (e) are in mm. Cylinders with radii of R = 20 mm (b) and (d) or R = 5 mm (c) and (e) are shown, each surrounded by a water-like material (low elastic moduli). Colors correspond to the amplitude of the shear waves, scaled by $r^{2}$ ( r is the distance from the source), to compensate for attenuation away from the source. One quadrant is shown; the other three are mirror images. Here, θ-motion amplitudes (in the y-z plane) are plotted.

**FIG. 2.**
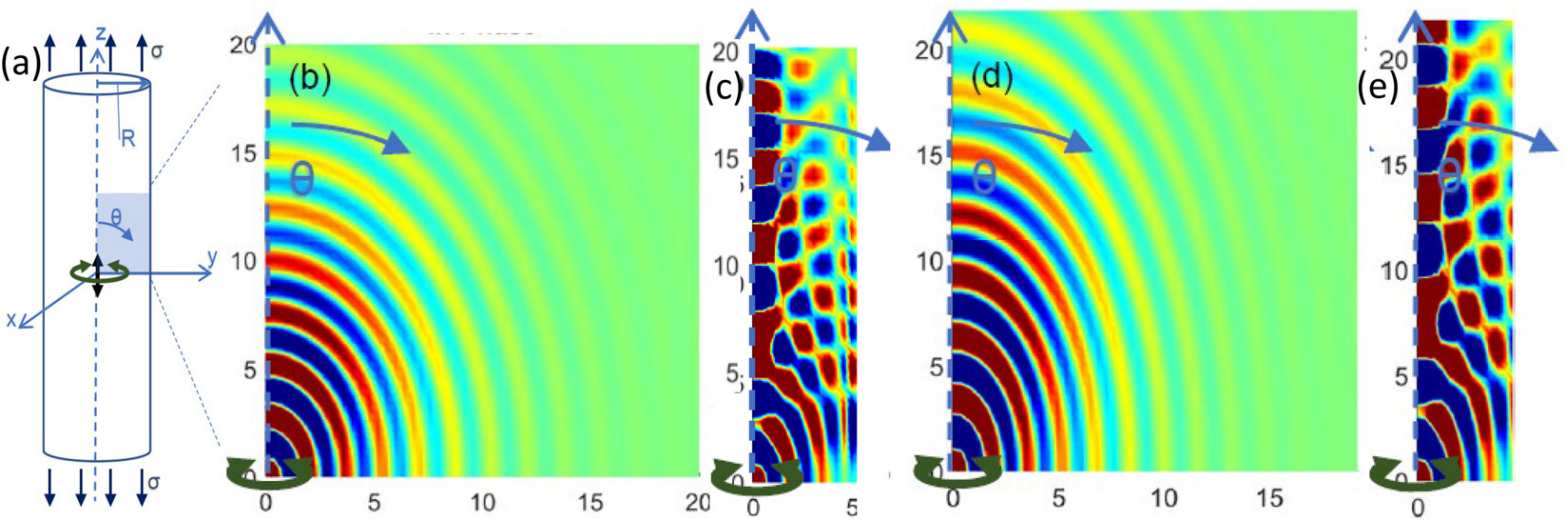
(Color online) Slow shear waves in the y-z plane generated by a harmonic point torque at the origin polarized orthogonal to the axis of isotropy (z-axis) and direction of uniaxial stress σ, using material properties from our previous work (Refs. 45 and 51–53) that correspond approximately to passive muscle with fibers along the z-axis (Table II). Simulations are for no pre-stress (b) and (c) or a uniaxial prestress from elongation along fiber direction that is equivalent to the stress from a 10% MVC contraction (d) and (e). All axes in (b)–(e) are in mm. Cylinders with radii of R = 20 mm (b) and (d) or R = 5 mm (c) and (e) are shown, each surrounded by a water-like material (low elastic moduli). Colors correspond to the amplitude of the shear waves, scaled by $r^{2}$ ( r is the distance from the source), to compensate for attenuation away from the source. One quadrant is shown; the other three are mirror images. Here, x-motion amplitudes are plotted.

Let us consider a point force or torque acting at the geometric center of a circular cylinder of a transversely isotropic (TI) material under a uniaxial stress σ aligned with both the axis of the cylinder and the axis of isotropy of the TI material, as shown in Figs. 1(a) and 2(a). For the case of plane wave propagation in this material, taking $\sigma_{x}=\sigma_{y}=0$ and taking $\sigma_{z}=\sigma$, slow and fast shear wave speeds squared, respectively, are

$${c(\theta )}_{s}^{2}=\frac{\mu_{⊥}}{\rho }\left(1+\phi \;{\text{cos}}^{2}\left[\theta \right]+\frac{\sigma }{2\mu_{⊥}}\;{\text{cos}}^{2}\left[\theta \right]\right),$$ (5)

$${c(\theta )}_{f}^{2}=\frac{\mu_{⊥}}{\rho }\left(1+\phi +\left(\zeta -\phi \right)\;{\text{sin}}^{2}\left[2\theta \right]+\frac{\sigma }{2\mu_{⊥}}\text{cos}\;\left[2\theta \right]\right).$$ (6)

Here, θ is the angle between the direction of propagation and the axis of isotropy and uniaxial stress.

A numerical integral solution for the response created for the unbounded case of a harmonically oscillating point force (infinitesimal dipole) in a TI material has been found,^50^ as well as analytical approximations to it.^51^ The authors are unaware if such a solution exists for the case with uniaxial stress or for the case of a point torque, or for any of these cases when the medium has finite boundaries. Consequently, for the following study, a numerical finite element (FE) approach is taken using ANSYS Mechanical APDL Version 2019 R1 (Ansys, Canonsburg, PA). The FE prediction was validated against the exact integral solution for the unstressed point force and taking the radius of the cylinder to be large enough that waves attenuate before reaching the boundary. The case study parameters shown in Table II are typical of soft biological tissue and match those used previously by the last author,^45,51–53^ but with the addition of cases with a nonzero uniaxial tensile stress σ (it is either 0 or equal to $\mu_{⊥R}$).

**TABLE II.** Parameter values for case studies.

Parameter	Nomenclature	Value(s)
Bulk modulus	κ	2.6 GPa
Shear storage modulus in plane of isotropy	$\mu_{⊥R}$	2.77 kPa
Ratio of shear loss to storage moduli	$\eta =\mu_{⊥I}\;/\mu_{⊥R}=\mu_{∥I}/\mu_{∥R}$	0.15
Shear anisotropy	ϕ	1
Tensile anisotropy	ζ	2
Uniaxial tensile stress/ $\mu_{⊥R}$	$\sigma /\mu_{⊥R}$	0 or 1
Density	ρ	1000 kg/m^3^
Frequency	f	1 kHz

First, a cylinder 70 mm in height and 40 mm in diameter was defined using an axisymmetric mixed u-P formulation with Plane183 8-node elements with individual element side dimensions of 0.1 mm. A point force was applied in the vertical “z” direction, parallel to the fiber and tensile load direction at the node located at the geometric center of the cylinder. Given the “z” polarization of the source input, it will predominantly drive fast shear waves. But, since this is a point source, the propagating wave field is not planar and thus, does not perfectly follow Eq. (2). The in phase steady state response for cases without and with a nonzero tensile load σ are shown in Figs. 1(b) and 1(d). Next, the point force is replaced by a point torque at the same location, oriented in the “z” direction, so that it predominantly drives waves that are polarized in the circumferential direction, which is orthogonal to the axis of isotropy and thus, behave like slow shear waves, approximately governed by Eq. (3). The in phase steady state response for cases without and with a nonzero tensile load σ are shown in Figs. 2(b) and 2(d).

In the simulations described above in this section, for the chosen material properties, cylinder diameter and excitation frequency, we see more than 8 wavelengths going from the excitation source (cylinder axis) to the cylinder free outer boundary at a radius of 20 mm. The effect of the free boundary is not apparent in this case where wavelength is an order of magnitude less than the characteristic cross-dimension. But consider this exact same case with the cylinder radius R reduced from 20 mm down to 5 mm, in other words, only a few wavelengths across. The simulations are shown in Figs. 1(c), 1(e), 2(c), and 2(e), identical to the cases in Figs. 1(b), 1(d), 2(b), and 2(d), respectively. While the wave pattern in the immediate vicinity of the source is similar, it has been substantially altered beyond a few wavelengths from the source. Differences caused by the presence of the uniaxial stress σ are still evident, but there are also complex wave patterns further from the source. These patterns are not described at all by Eqs. (5) and (6), although a modified form (to account for σ) of the equations in Sec. 8.2.2 of Graff^54^ might apply.

## IV. TWO-DIMENSIONAL WAVEGUIDE WITH IN-PLANE BIAXIAL NORMAL STRESS

### A. Theory

**FIG. 3.**
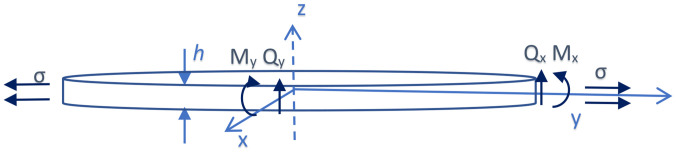
(Color online) Two-dimensional waveguide.

Next, we will consider a thin plate of thickness h in the z-direction, with z still the axis of isotropy, but take: $\sigma_{x}=\sigma_{y}=\sigma$ and take $\sigma_{z}=0$, as shown in Fig. 3. We will focus on shear waves in the x-y plane that are polarized in the z direction. In an unbounded three-dimensional medium, based on Eqs. (2)–(4) and Table I, the phase speed of plane waves propagating in the x-y plane with z polarization are

$$c_{f}^{2}=\frac{\mu_{⊥}}{\rho }\left(1+\phi +\frac{\sigma }{2\mu_{⊥}}\right).$$ (7)

However, we will employ the following assumptions based on Mindlin's first contribution that follow from assuming that plate thickness h is less than the shear wavelengths of interest (Sec. 8.3 of Ref. 54): $u\left[x,y,z,t\right]=z\varphi_{x}\left[x,y,t\right],v\left[x,y,z,t\right]=z\varphi_{y}\left[x,y,t\right],\;w\left[x,y,z,t\right]=\bar{w}\left[x,y,t\right]$. Furthermore, we will assume that $\varphi_{x}=-w_{,x}\;\;\mathrm{and}\;\;\varphi_{y}=-w_{,y}$, which is consistent with Euler–Bernoulli thin-plate theory. Utilizing Eq. (2.1) of Ref. 47, and inputting the above assumptions:

$$\sigma_{xx,x}+\sigma_{xy,y}+\sigma_{xz,z}-\sigma \frac{1}{2}\left(u_{,zz}\right)=\sigma_{xx,x}+\sigma_{xy,y}+\sigma_{xz,z}=\rho u_{,tt},$$ (8)

$$\sigma_{yx,x}+\sigma_{yy,y}+\sigma_{yz,z}-\sigma \frac{1}{2}\left(v_{,zz}\right)=\sigma_{yx,x}+\sigma_{yy,y}+\sigma_{yz,z}=\rho v_{,tt},$$ (9)

$$\begin{array}{c} \sigma_{zx,x}+\sigma_{zy,y}+\sigma_{zz,z}+\sigma \frac{1}{2}\left(w_{,xx}-u_{,zx}+w_{,yy}-v_{,zy}\right)\\ =\sigma_{zx,x}+\sigma_{zy,y}+\sigma_{zz,z}+\sigma \left(w_{,xx}+w_{,yy}\right)=\rho w_{,tt}. \end{array}$$ (10)

Adapting Sec. 8.3 of Ref. 54, we multiply Eqs. (8) and (9) by *z* and integrate across the plate thickness from −h/2 to+h/2, and we directly integrate Eq. (10) across the plate thickness from −h/2 to+h/2. Neglecting higher order terms, this leads to

$$M_{x,x}+M_{yx,y}-Q_{x}+\rho \frac{h^{3}}{12}w_{,\mathrm{xtt}}=0,$$ (11)

$$M_{xy,x}+M_{y,y}-Q_{y}+\rho \frac{h^{3}}{12}w_{,\mathrm{ytt}}=0,$$ (12)

$$Q_{x,x}+Q_{y,y}+\sigma h\left(w_{,xx}+w_{,yy}\right)+q=\rho hw_{,tt},$$ (13)

where $M_{x},\;M_{y},\;M_{xy},\;\text{and}\;M_{yx}$ are bending moments about the plate, $Q_{x}$ and $Q_{y}$ are shear forces, and q is an externally applied force per unit area to the plate (Fig. 3). Expressions for the bending moments are^55^

$$M_{x}=\frac{E_{⊥}h^{3}}{12\left(1-\nu_{xy}^{2}\right)}\left(\varphi_{x,x}+\nu_{xy}\varphi_{y,y}\right)=-\frac{E_{⊥}h^{3}}{12\left(1-\nu_{xy}^{2}\right)}\left(w_{,xx}+\nu_{xy}w_{,yy}\right),$$ (14)

$$M_{y}=\frac{E_{⊥}h^{3}}{12\left(1-\nu_{xy}^{2}\right)}\left(\varphi_{y,y}+\nu_{xy}\varphi_{x,x}\right)=-\frac{E_{⊥}h^{3}}{12\left(1-\nu_{xy}^{2}\right)}\left(w_{,yy}+\nu_{xy}w_{,xx}\right),$$ (15)

$$M_{xy}=M_{yx}=\frac{E_{⊥}h^{3}}{24\left(1+\nu_{xy}\right)}\left(\varphi_{y,x}+\varphi_{x,y}\right)=-2\frac{\mu_{⊥}h^{3}}{12}\left(w_{,xy}\right).$$ (16)

Neglecting rotational inertia terms in Eqs. (11) and (12) since h is small, taking partial derivatives with respect to x and y, respectively, solving for $Q_{x,x}$ and $Q_{y,y}$ and substituting into Eq. (13) leads to the following:

$$M_{x,xx}+2M_{xy,yx}+M_{y,yy}+\sigma hw_{,xx}+\sigma hw_{,yy}+q=\rho hw_{,tt}.$$ (17)

This can be written as

$$Dw_{,\mathrm{xxxx}}+2D_{xy}w_{,\mathrm{xxyy}}+Dw_{,\mathrm{yyyy}}+\rho hw_{,tt}=\sigma hw_{,xx}+\sigma hw_{,yy}+q,$$ (18)

where

$$D=\frac{E_{⊥}h^{3}}{12\left(1-\nu_{xy}^{2}\right)},$$ (19a)

$$D_{xy}=D\nu_{xy}+\frac{\mu_{⊥}h^{3}}{12}.$$ (19b)

Neglecting external normal load per unit area q and assuming a planar wavefront in the *x* direction, partial derivatives with respect to y lead to zero and the equation simplifies to the following:

$$Dw_{,\mathrm{xxxx}}+\rho hw_{,tt}=\sigma hw_{,xx}.$$ (20)

The general solution to Eq. (20) for harmonic motion is given in the form $w=We^{i(\gamma x-\omega t)}$, leading to the following set of solutions:

γ=±α, ±iβ, (21a)

$$\alpha ={\left\{-\xi +{\left(\xi^{2}+\frac{\omega^{2}}{a^{2}}\right)}^{1/2}\right\}}^{1/2},$$ (21b)

$$\beta ={\left\{\xi +{\left(\xi^{2}+\frac{\omega^{2}}{a^{2}}\right)}^{1/2}\right\}}^{1/2},$$ (21c)

$$\xi =\frac{\sigma h}{2D},$$ (21d)

$$a=\sqrt{\frac{D}{\rho h}}.$$ (21e)

Thus, we have two propagating waves (α) in the + or – x direction, and two non-propagating (near field or evanescent) waves (β) in the + or – x direction. For the propagating waves, the phase speed will be: $c_{ph}=\omega /\mathrm{Real}\left[\alpha \right]$. Taking the limit that σh≪2D, we see that $\alpha ={\left(\omega /a\right)}^{1/2}$ and thus, for the elastic case $c_{ph}=\omega^{1/2}{\left(D/\rho h\right)}^{1/4}$. Note, there is dispersion even in the lossless elastic case (neglecting viscosity); this is the classic unstressed thin transverse plate vibration solution. On the other hand, taking the limit of tension σh≫2D, we then drop $Dw_{,\mathrm{xxxx}}$ from Eq. (20) and reformulate the solution to find there are two propagating solutions in the + or – x direction with $c_{ph}={\left(\sigma /\rho \right)}^{1/2}$. This is the classic thin membrane vibration solution. Note, none of these solutions match the expression for shear wave propagation under the same conditions but with h very large (3-dimensional medium) which is provided in Eq. (7).

### B. Application to cornea elastography

The eye can be modeled as an internally pressurized spherical vessel where an increase in the internal pressure strains the walls of the ocular tissue and induces a tensile, circumferential hoop stress $\left(\sigma_{\theta }\right)$. The range of tensile stress can be roughly estimated using Laplace's law for a spherical pressure vessel as $\sigma_{\theta }=Pr/2h$, where P is the intraocular (gage) pressure (IOP), r is the radius of the sphere, and h is the thickness of the sphere walls. By assuming a radius of 10 mm and thickness of 0.6 mm, IOP values of 0, 5, 10, 15, and 20 mmHg will induce hoop stresses of 0, 5.56, 11.11, 16.67, and 22.22 kPa, respectively. For comparison, finite element (FE) studies using more precise geometry have reported hoop stresses within the range of roughly 15–25 kPa for around 15–18 mmHg IOP.^56,57^

**FIG. 4.**
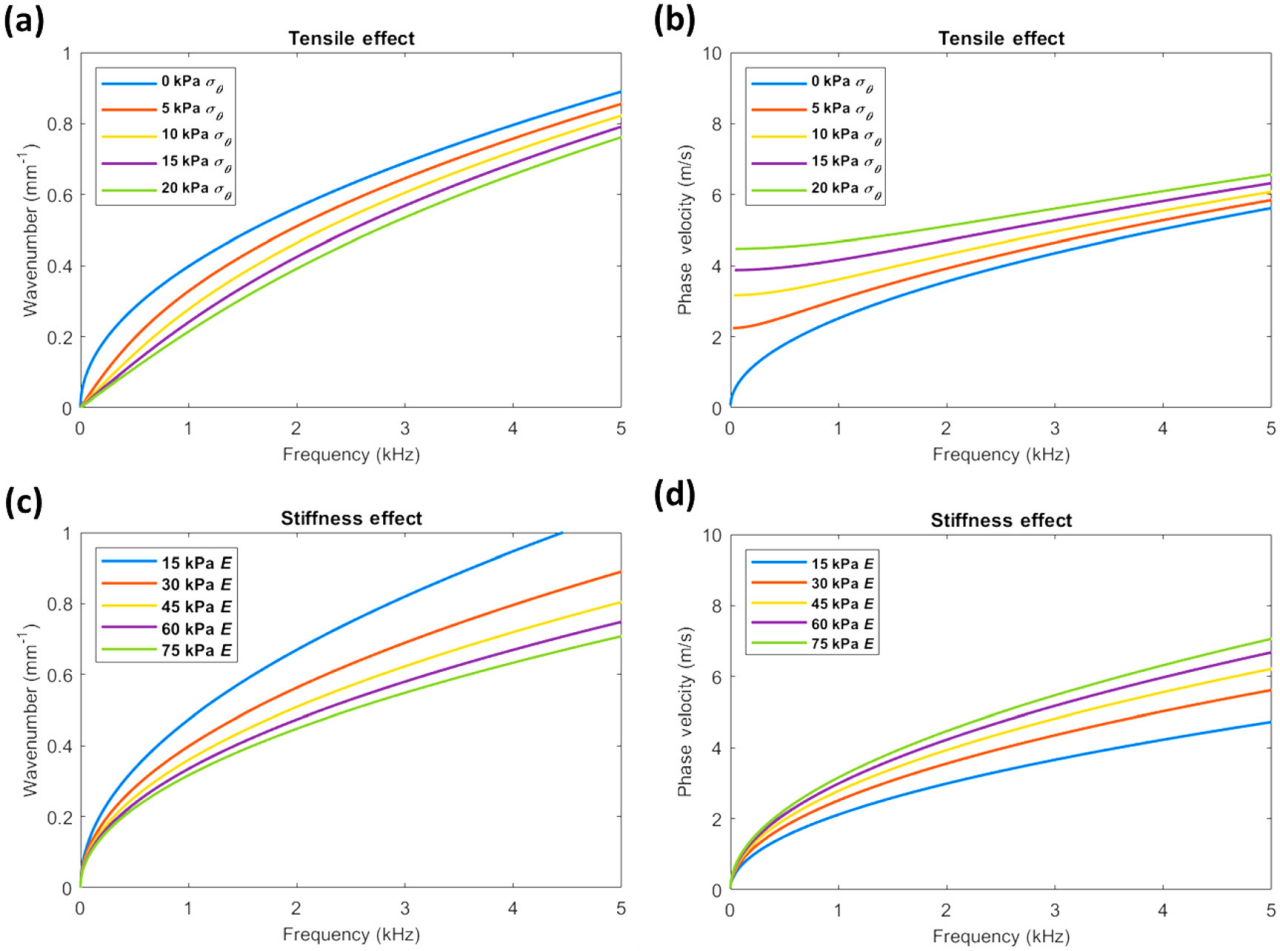
(Color online) Thin-plate theory for comparing the effects of tensile prestress and stiffness on wave dispersion. (a) The linear wavenumber vs frequency dispersion curves demonstrate that estimated prestress values $\sigma_{\theta }$ incremented between 0 and 2.0 × 10^4^ Pa can alter wave behavior independently of material stiffness (where $E_{⊥}$ = 3.0 × 10^4^ Pa). (b) The corresponding phase velocity curves demonstrate that tension strongly influences low-frequency phase velocities while high-frequency waves are also affected. (c) Dispersion curves demonstrate that stiffness affects wave behavior similar to tension when incremented between 1.5 and 7.5 × 10^4^ Pa (where $\sigma_{\theta }$ = 0 Pa), though its effects at high frequencies are more apparent. Additionally, when comparing (a) and (c), the low-frequency components below ∼1 kHz are affected less by stiffness than tension. (d) This is reflected in the corresponding phase velocity curves where low frequency phase velocities are less influenced by stiffness. Further comparison of (b) and (d) show that both tension and stiffness can change the high-frequency phase velocities that are used to estimate corneal stiffness.

To evaluate whether tensile in-plane biaxial pre-stress, being equated to hoop stress, within this range can influence transverse wave motion on the cornea, we use equations from Sec. IV A with $\rho =1\;g/cm^{3}$, h=0.6 mm, $E_{⊥}=50\;\mathrm{kPa}$ and $\nu_{xy}=0.499$, yielding $D=1.2\times 10^{-6}\;Nm$. Pre-stress was incremented between 0 and 20 kPa to estimate the tensile effect of IOP on wavenumber [Fig. 4(a)] and phase speed [Fig. 4(b)]. For comparison, Young's modulus $E_{⊥}$ was also separately incremented between 15 and 75 kPa, with zero hoop stress [Figs. 4(c) and 4(d)].

Simulation results shown in Fig. 4 suggest that increasing both the tensile pre-stress (via increasing the IOP) and Young's modulus (a material property) can alter phase speed similarly. The slope of the dispersion curves decreases as both tension and material stiffness increase which corresponds to an increase in phase velocities. Importantly, this indicates that the high frequency asymptotes of the dispersion curves (above ∼3 kHz) that are normally used to estimate corneal shear moduli can be significantly influenced by both pre-stress and Young's modulus. While both effects are similar, differences in the dispersion curves can be observed, particularly in the lower frequency regions (below ∼1 kHz) from the approximate thin-plate model; tensile pre-stress appears to strongly influence the low-frequency dispersion behavior, while the effects of stiffness are more apparent at higher frequencies. These results suggest that tensile pre-stresses within the range predicted for normal and pathological IOP values can indeed influence wave behavior and that differential analysis of low- and high-frequency dispersion components may help to discern the effects of pre-stress and stiffness. It appears that if these pre-stress effects are not accounted for, an overestimation of corneal shear moduli may arise.

In Sun *et al.* (2021),^58^ analysis of the effect of tensile pre-stress and a foundation stiffness is further explored by integrating models for them into the full three-dimensional theory introduced in Sec. II. Trends identified using the thin plate theory are consistent with what is found using the more precise model, in that failure to account for the effects of pre-stress may result in overestimations of the corneal elastic moduli, particularly at high IOPs.

## V. ONE-DIMENSIONAL WAVEGUIDE WITH UNIAXIAL NORMAL STRESS

### A. Theory

Starting with the transverse isotropic material model introduced in Sec. II, consider a rod whose axis is aligned with the z-axis, the axis of isotropy, with cross-sectional dimensions in the x and y direction that are significantly less than wavelength. In other words, take the numerical study of Sec. III but with *r* smaller. Also, as in Sec. III, set $\sigma_{x}=\sigma_{y}=0$ and $\sigma_{z}=\sigma$. Undertaking an analysis similar to Sec. IV, we arrive at the pre-tensioned Euler–Bernoulli thin beam described in Section 3.3.4 of Graff^54^ for x-polarized transverse wave propagation of the beam along its z-axis,

$$E_{∥}Iu_{,\mathrm{zzzz}}-\sigma Au_{,zz}+A\rho u_{,tt}=0.$$ (22)

Here, I is the area moment of inertia about the *y* axis ( $I=\frac{\pi }{4}r^{4}$ for a circular cross section of radius r), and A is the cross-sectional area in the x-y plane. Note the similarity to the Eq. (20) of Sec. IV.

Consider this an initial approximation for a thin muscle under a tensile load along its axis. As before, the general solution form is: $u=Ue^{i(\gamma x-\omega t)}$, where $i=\sqrt{-1}$, ω is the circular frequency in rad/s and γ has four possible solutions,

γ=±α, ±iβ, (23a)

$$\alpha ={\left\{-\xi +{\left(\xi^{2}+\frac{\omega^{2}}{a^{2}}\right)}^{1/2}\right\}}^{1/2},$$ (23b)

$$\beta ={\left\{\xi +{\left(\xi^{2}+\frac{\omega^{2}}{a^{2}}\right)}^{1/2}\right\}}^{1/2},$$ (23c)

$$\xi =\frac{\sigma A}{2E_{∥}I},$$ (23d)

$$a=\sqrt{\frac{E_{∥}I}{\rho A}}.$$ (23e)

Like in Sec. IV A, we have two propagating waves (α) in the + or – x direction, and two non-propagating (near field or evanescent) waves (β) in the + or – x direction. For the propagating waves, the phase speed will be: $c_{ph}=\omega /\mathrm{Real}\left[\alpha \right]$. Taking the limit that $\sigma A\ll 2E_{∥}I,$ we see that $\alpha ={\left(\omega /a\right)}^{1/2}$ and thus for the elastic case $c_{ph}=\omega^{1/2}{\left(E_{∥}I/\rho A\right)}^{1/4}$, which is the classic thin (Euler–Bernoulli) beam transverse vibration solution. On the other hand, taking the limit of tension $\sigma A\gg 2E_{∥}I,$ we then drop $E_{∥}Iu_{,\mathrm{zzzz}}$ from Eq. (22) and reformulate the solution to find that there are two propagating solutions with $c_{ph}={\left(\sigma /\rho \right)}^{1/2}$. This is the classic transverse thin string vibration solution. As noted before in Sec. IV A, a case between the extremes of either neglecting σA or $E_{∥}I$ still does not match the phase speed of bulk shear waves, which again is given by Eq. (7).

### B. Application to muscle elastography

In a recent study of shear wave elastography on an *ex vivo* cat soleus by Bernabei *et al.*,^59,60^ where the tensile preload σA could be precisely measured while conducting elastography measurements on a passively tensioned muscle as well as when it is activated via an electrical current, it was found that under passive tensile loading, the wave speed closely followed the string solution with $c_{ph}={\left(\sigma /\rho \right)}^{1/2}$. However, under muscle activation phase speed appeared to fall between the two extreme cases articulated above (Sec. V A). Other studies have shown similar trends,^24–30^ but have ascribed the increase in $c_{ph}$ to be due to an increase in the elastic moduli of the muscle, in other words its nonlinearity under deformation. The cat soleus has a physiological cross sectional area (PCSA) of about 1 cm^2^, or a diameter of about 1 cm. Human muscles studied using elastography can have a wide range of PCSAs, for example, as small as 0.68 cm^2^ in the abductor pollicis brevis^61^ to 21 cm^2^ in the medial gastrocnemius.^62^

## VI. CONCLUDING REMARKS: NONLINEARITY AND THE GLASS HALF FULL

The experimental method of elastography generally involves small oscillatory deformations and it is reasonable to assume linear theory is valid. However, the static deformations, due to pre-stress in the applications described above (Secs. IV and V), may be significant and not be reasonably modeled based solely on linear systems theory. A logical approach is to apply a more robust nonlinear theory to model the static deformation created by the pre-stress using an appropriate strain energy function, followed by linearization about the new static equilibrium in order to derive an elasticity matrix that can be used for the “linearized” *acoustoelastography* problem. A few groups in the elastography community have been investigating this and identify higher order elastic constants. This remains an active area of research with a wide range of strain energy functions having been proposed, with it proving difficult to experimentally evaluate which may be most appropriate for a given application.^26,48,49,63–65^

Even without the added complexity of geometric and material nonlinearities due to large static deformation, the elastography problem, especially in anisotropic materials, is made more challenging by the presence of static or quasi-static stress loads that are inherent to the normal physiological function of many biological soft tissues. They cannot be avoided. So is the increase in phase speed as a tissue is further stretched caused by the stretching (tensile load) itself, or is it caused by the higher order elasticity coefficients needed to fully characterize the material nonlinearity of the tissue over a large deformation range? Can these effects be teased apart through careful experimental measurements that fully investigate all manner of direction and polarization of mechanical wave motion at multiple static deformation levels? Are such experiments feasible? Recognition of these daunting technical challenges may lead one to think that the technique of elastography is, indeed, a half empty glass, unable to confidently determine that which it originally set out to determine, a material's elastic constants. But this would be missing the forest for the trees. Being clear-eyed about the challenges that we face, we believe the glass is half full, in that once we overcome the known complexities of the problem, *acoustoelastography* will be able to noninvasively quantitatively map not only a material's complex stiffness, but also the complex stress field it is under. Stiffness and stress are both critical to the physiological function of many biological tissues, and both can be uniquely altered by pathology, injury, and response to therapy. A tool that can nondestructively measure both independently would be profound.

## APPENDIX

Infinitesimal strain theory based on linear elasticity in a transverse isotropic (TI) material culminates in the Eqs. (2)–(4) of motion and the phase speed values given in Table I and Eqs. (5) and (6). This approach does not account for changes brought about by the altered geometry due to the deformation or by any nonlinearity in the material properties. Other studies have started from the more general non-linear theory of a hyperelastic material with initial stress, defining a finite strain energy function, from which linear and higher order elastic coefficients can be derived for study of wave propagation.^26,48,49,63–65^ In Destrade *et al.*^48^ and Remenieras *et al.*^49^ elastic wave phase speed in an incompressible TI solid under uniaxial stress was considered by starting with a third-order expansion of the elastic strain energy function given in powers of the Green–Lagrange strain tensor. For the case that is the same as the uniaxial formulation in the current study (uniaxial stress direction aligned with the axis of isotropy), the following equations were derived for the slow $c_{s}$ and fast $c_{f}$ shear wave phase speeds, identifying the relationships between the nomenclature of the referenced studies to the current study:

$${c\left[\theta \right]}_{s}^{2}=\frac{\mu_{⊥}}{\rho }\left(1+\phi \;{\text{cos}}^{2}\left[\theta \right]+\beta_{∥}\frac{\sigma }{\mu_{⊥}}\;{\text{cos}}^{2}\left[\theta \right]-\beta_{⊥}\frac{\sigma }{\mu_{⊥}}\;{\text{sin}}^{2}\left[\theta \right]\right),$$
(A1)

$${c\left[\theta \right]}_{f}^{2}=\frac{1}{\rho }\left(\left(\alpha +\gamma -2\beta \right)\;{\text{cos}}^{4}\left[\theta \right]+2\left(\beta -\gamma \right)\;{\text{cos}}^{2}\left[\theta \right]+\gamma \right),$$
(A2)

where

$$\beta_{∥}=1+\frac{1}{E_{∥}}\left(\mu_{⊥}\phi +\frac{A}{4}+\alpha_{3}+\frac{\alpha_{5}}{2}\right)=1+\frac{\phi }{3+4\zeta }+\frac{1}{\mu_{⊥}\left(3+4\zeta \right)}\left(\frac{A}{4}+\alpha_{3}+\frac{\alpha_{5}}{2}\right),$$
(A3)

$$\beta_{⊥}=\frac{1}{E_{∥}}\left(3\mu_{⊥}+\frac{A}{2}-\alpha_{3}\right)=1-\frac{4\zeta }{3+4\zeta }+\frac{1}{\mu_{⊥}\left(3+4\zeta \right)}\left(\frac{A}{2}-\alpha_{3}\right),$$
(A4)

$$\alpha +\gamma -2\beta =-4\mu_{⊥}\left(\zeta -\phi \right)-2\left(8\mu_{⊥}\left(\zeta -\phi \right)+6\mu_{⊥}\phi +3\alpha_{3}+3\alpha_{4}+2\alpha_{5}\right)\frac{\sigma }{\mu_{⊥}\left(3+4\zeta \right)},$$
(A5)

$$2\left(\beta -\gamma \right)=4\mu_{⊥}\left(\zeta -\phi \right)+\left(3\mu_{⊥}+20\mu_{⊥}\left(\zeta -\phi \right)+16\mu_{⊥}\phi +6\alpha_{3}+6\alpha_{4}+4\alpha_{5}\right)\frac{\sigma }{\mu_{⊥}\left(3+4\zeta \right)},$$
(A6)

and

$$\gamma =\mu_{⊥}\left(1+\phi \right)+\frac{1}{4}\left(A+4\mu_{⊥}\phi +4\alpha_{3}+2\alpha_{5}\right)\frac{\sigma }{\mu_{⊥}\left(3+4\zeta \right)}.$$
(A7)

Here, A is the third-order constant of weakly nonlinear incompressible isotropic elasticity^48,63^ and $\alpha_{3}$, $\alpha_{4}$, and $\alpha_{5}$ are additional anisotropic third-order constants present in the transverse isotropy case.^48,49^

Ogden and Singh^66^ showed that the formulation provided by Biot's linear elasticity theory,^46^ which Eqs. (5) and (6) in the present study are based upon, leads to $A=-6\mu_{⊥}\left(1+\phi \right)$, $\alpha_{3}=-3\mu_{⊥}\phi$, $\alpha_{4}=-\mu_{⊥}\phi$, and $\alpha_{5}=\mu_{⊥}\left(7\phi -4\zeta \right)$. Placing these values in Eqs. (A1) and (A2) results in $\beta_{∥}=\frac{1}{2}$, $\beta_{⊥}=0,\;\gamma =\mu_{⊥}\left(1+\phi \right)-\sigma /2,\;\beta =\mu_{⊥}\left(1-\phi +2\zeta \right),$ and $\alpha =\mu_{⊥}\left(1+\phi \right)+\sigma /2$. Then, Eqs. (A1) and (A2) will exactly match Eqs. (5) and (6).

Values for the parameter A have been estimated experimentally in tissue-like isotropic materials. For example, Gennisson *et al.*^26^ and Urban *et al.*^67^ found a wide range of estimated values for A relative to $\mu_{⊥}$ [encompassing $A=-6\mu_{⊥}(1+\phi )$] by measuring shear wave speeds in different directions and polarizations relative to the axis of uniaxial compressive stress in agar, gelatin, agar-gelatin, and polyvinyl alcohol phantoms with compressive stresses up to and exceeding 10% of the measured $\mu_{⊥}$. These studies also found that strain levels, even as low as 1%, resulted in a measured nonlinear relationship between strain and small amplitude shear wave speed, thus requiring at least fourth order elasticity constants that have a quadratic dependence on strain (or stress). Thus, we acknowledge the limitations of the current analysis based on linear elasticity, with specified third order elasticity constants and no fourth or higher order constants that are necessary to model material nonlinearities. Nonetheless, the simplified analysis here has illustrated the importance of accounting for stress in elastography measurements with and without the additional complexity of finite boundaries.
